# Genomic patterns of nucleotide diversity in divergent populations of U.S. weedy rice

**DOI:** 10.1186/1471-2148-10-180

**Published:** 2010-06-15

**Authors:** Michael Reagon, Carrie S Thurber, Briana L Gross, Kenneth M Olsen, Yulin Jia, Ana L Caicedo

**Affiliations:** 1 Biology Department, University of Massachusetts, Amherst, MA 01003, USA; 2 Department of Biology, Washington University, St. Louis, MO 63130, USA; 3 USDA-ARS Dale Bumpers National Rice Research Center, Stuttgart, AR 72160, USA

## Abstract

**Background:**

Weedy rice (red rice), a conspecific weed of cultivated rice (*Oryza sativa *L.), is a significant problem throughout the world and an emerging threat in regions where it was previously absent. Despite belonging to the same species complex as domesticated rice and its wild relatives, the evolutionary origins of weedy rice remain unclear. We use genome-wide patterns of single nucleotide polymorphism (SNP) variation in a broad geographic sample of weedy, domesticated, and wild *Oryza *samples to infer the origin and demographic processes influencing U.S. weedy rice evolution.

**Results:**

We find greater population structure than has been previously reported for U.S. weedy rice, and that the multiple, genetically divergent populations have separate origins. The two main U.S. weedy rice populations share genetic backgrounds with cultivated *O. sativa *varietal groups not grown commercially in the U.S., suggesting weed origins from domesticated ancestors. Hybridization between weedy groups and between weedy rice and local crops has also led to the evolution of distinct U.S. weedy rice populations. Demographic simulations indicate differences among the main weedy groups in the impact of bottlenecks on their establishment in the U.S., and in the timing of divergence from their cultivated relatives.

**Conclusions:**

Unlike prior research, we did not find unambiguous evidence for U.S. weedy rice originating via hybridization between cultivated and wild *Oryza *species. Our results demonstrate the potential for weedy life-histories to evolve directly from within domesticated lineages. The diverse origins of U.S. weedy rice populations demonstrate the multiplicity of evolutionary forces that can influence the emergence of weeds from a single species complex.

## Background

Among the most widespread and costly agricultural pests are the numerous weeds that have evolved from within the same complex of interfertile species as domesticated plants [1-3]. The recent and rapid evolution of these conspecific weeds also presents unique opportunities to study processes influencing adaptive population divergence and parallel evolution of weedy life-histories. Conspecific weeds are morphologically and ecologically divergent from domesticated and wild congener species, and are not simply transient "volunteers" of the previous season's crop [4,5]. The evolutionary success of conspecific weeds is often attributed to acquisition of traits associated with wild plants (e.g. dormancy), presumably selected against in crops. Conversely, these weeds also often exhibit characteristics typical of domesticated plants, (e.g. more selfing, rapid growth), which could promote invasiveness in the agroecosystem. There is great interest in understanding the evolutionary mechanisms that can lead to the emergence of weedy species from the same species complexes that give rise to domesticated plants.

The larger complex of interfertile species within which conspecific weeds evolve includes the crop, wild relatives, and other feral weeds [6]. Studies have shown that, in many cases, hybridization between crops and wild species can facilitate weed evolution [reviewed in [7,8]]. Alternatively, conspecific weeds may evolve from standing genetic variation in wild relatives [7], or cultivated germplasm [e.g. [9]], though examples of weeds evolving directly from crops are rare. The short evolutionary time scales involved make it less likely that novel mutations are significant to weed evolution, however exceptions are known [e.g. [10]].

Here we investigate the evolutionary origins of weedy rice in the United States, which has been a subject of considerable debate for more than 150 years [11-16]. Weedy or red rice (due to the frequent presence of a red pericarp), is found in cultivated rice fields worldwide, but is most damaging in direct seeded (seeding directly into a dry soil bed), highly mechanized agricultural systems typical of the U.S., Europe and Australia [17]. Although currently classified as the same species as Asian cultivated rice, *Oryza sativa *L., weedy rice has morphological characteristics typical of wild species (e.g. dormancy, shattering) and of cultivated rice (e.g. high fecundity, high selfing rate). The long term persistence of weedy rice throughout the range of cultivated rice, suggests that it can adapt to local changes in agronomic practices as well as different biotic and abiotic conditions [18,19].

**Figure 1:**
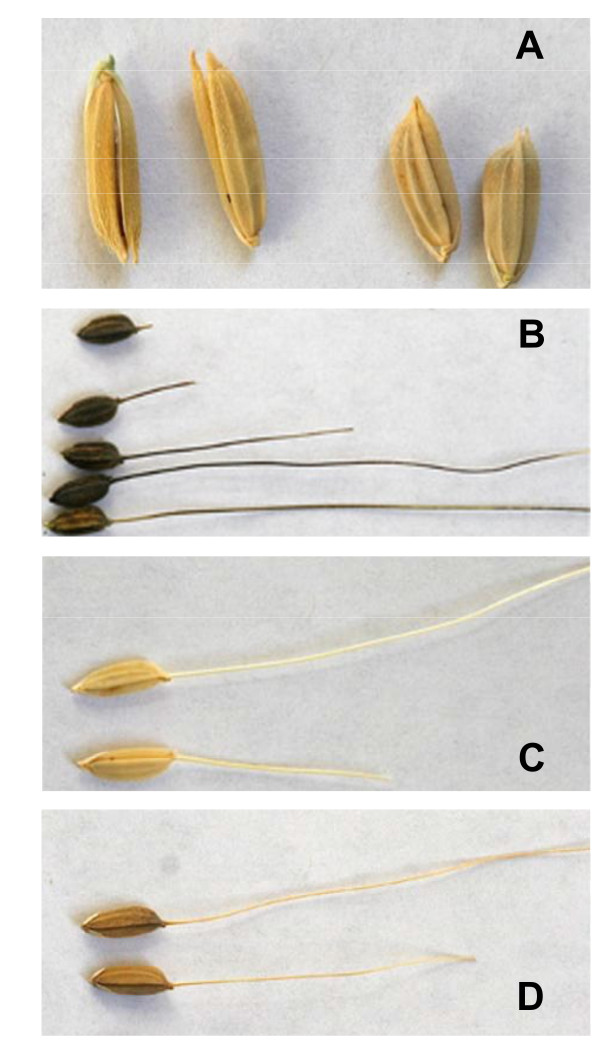
**Hull morphologies of U.S. weedy rice**. A) straw hull no awns; this morphology is typical of the SH population (see text for explanation), B) black hull with awns; this morphology is typical of the BHA1 and BHA2 populations (see text for explanation), C) straw hull with awns; this morphology is also found in BHA1 and BHA2 populations, D) brown or striped hull with awns: this morphology is typical of the BRH population (see text for explanation)

No *Oryza *is native to the U.S.; therefore, U.S. weedy rice must have evolved elsewhere and/or endogenously from introduced cultivated and/or wild germplasm. The *Oryza *crop-wild complex, within which weedy rice evolved, is composed of two domesticated and six wild species that share the AA genome [20,21]. Evidence for gene flow among members of this complex is extensive [16,22-26], suggesting that any of these taxa could have contributed to the origins of weedy rice in the U.S. The earliest available reference to weedy rice in the U.S. dates from 1846 [11], and describes a well-established and troublesome pest. Considerable phenotypic diversity is found within U.S. weedy rice populations [see references in [17]]. Currently, two main morphological groups include awnless straw-hulled types, which more closely resemble cultivated rice varieties, and awned black-hulled forms, with other morphologies found less frequently (Figure 1). Several SSR and RAPD studies have suggested that strawhull, awnless weedy rice is most closely related to *indica*, *O. sativa *varieties typical of lowland tropical regions, and probably a product of hybridization with *O. rufipogon*/*O. nivara*, the wild ancestor of domesticated Asian rice [13-15,27]. A recent microsatellite study suggests that some black hull, awned weedy rice may be derived from *O. sativa aus *varieties [13], a group most commonly grown in Bangladesh and Northeastern India, or from *O. rufipogon *[14]. To date, however, patterns of DNA sequence diversity have not been explored in U.S. weedy rice, and open questions remain about the likelihood of weed origins from cultivated ancestors, and the roles of demographic history and hybridization in the evolution of weedy rice.

Taking advantage of the existing genomic resources for domesticated rice [28,29], we use genome-wide patterns of DNA sequence variation in a broad sample of the *Oryza *crop-wild complex, to infer the origin and demographic history of U.S. weedy rice. Specifically we attempt to address remaining uncertainties regarding 1) the ancestral *Oryza *group(s), including other wild species, that gave rise to U.S. weedy rice, 2) the timing of divergence between U.S. weedy rice and its progenitor(s), and, 3) the role of hybridization in the establishment of U.S. weedy rice populations. We find considerable population structure in U.S. weedy rice, with genetically divergent populations having separate origins. Exotic cultivated *O. sativa *varieties are the main contributors to weedy rice genomes, and there is little evidence of contribution from wild *Oryza*. Hybridization among weedy groups has also influenced the emergence of novel weed phenotypes. Assessments of demographic parameters suggest differences among divergent weedy groups in the effect of population bottlenecks upon U.S. colonization, and in the timing of their origins. Our results demonstrate how similar weedy life histories can evolve from divergent genetic backgrounds.

## Methods

### Plant material

#### Weedy rice

Weedy rice seed was obtained from collections made over a period of 30 years in the Southern rice belt (Arkansas, Louisiana, Mississippi, Missouri and Texas) and maintained by the United States Department of Agriculture (USDA) at the Dale Bumpers Rice Research Institute, Stuttgart Arkansas (Additional file 1). We selected a subset of 58 accessions that maximized geographical diversity, but were otherwise chosen at random. We also included a few samples representative of rare morphologies (i.e. brown hulls), to increase the probability of capturing all existing population structure. Accessions listed in Additional file 1 as single seed descent are derived from seeds collected at rice mills and have been selfed at the USDA for four generations (D. Gealy personal communication). The remaining accessions were collected directly from weedy plants occurring in cultivated rice fields by the USDA.

#### Putative parental populations

For our analyses, we used data from 206 *Oryza *accessions, 95 of which were included in [30], and 111 which were chosen specifically for this study. Our sample broadly surveys AA genome *Oryza *species for potential parental sources of U.S. weedy rice (Additional file 2). We included Asian landraces and modern accessions from the five main variety groups of *O. sativa*; this includes 22 *indica*, 7 *aus*, 18 *tropical japonica *(varieties grown in tropical and subtropical regions), 22 *temperate japonica *(varieties typical of northern latitudes), and 6 *aromatic *(fragrant rice varieties). A plurality of evidence supports the independent domestication of the *indica *and *aus *groups from the *japonica *and *aromatic *groups beginning ~ 10,000 ybp from divergent populations of *O. rufipogon *[see [31]] (for alternate views see [32,33]). An additional 12 *tropical japonica *cultivars were added that are representative of important U.S. founding lineages (i.e. Carolina Gold, Blue Rose; [34]) or have been extensively grown in the southern U.S. We included 50 *O. rufipogon *and 3 *O. nivara *(a species often considered an annual form of *O. rufipogon *[35]) accessions, sampled across their geographic range. More samples from India and China were included as these regions are the possible centers of origin for domesticated rice [20,36]. Four accessions of African domesticated rice (*O. glaberrima*) and three of its wild progenitor (*O. barthii*) were included, as historical evidence suggests their introduction by early crop breeders and Africans brought to the U.S. as slaves [37]. Similarly, two accessions of *O. glumaepatula *were included, as it occurs in the Caribbean and Central America, and may have contributed to the evolution of weedy rice. *O. meridionalis*, native to Australia and Oceania, was included as an outgroup, as phylogenetic evidence indicates that it is ancestral to other AA genome *Oryza *[38].

### DNA extraction and sequencing

DNA was extracted from approximately 1 g of fresh leaf material from one plant per accession using a modified CTAB protocol [39,40]. DNA concentrations were gel quantified and diluted to 2 ng/ul for sequencing. We amplified and sequenced a total of 48, ~ 400-600 bp, gene fragments, selected from a set of 111 randomly chosen sequenced tagged loci (STS) developed by [30]. The 48 fragments were chosen to include ~4 loci per chromosome distributed on both chromosome arms (Additional file 3), without referencing diversity data or estimates of informativeness [41].

DNA sequencing was carried out in Cogenics sequencing facilities (Houston, TX) as described in [30,42]. Base pair calls, quality score assignment and construction of contigs were carried out as described in [30]. Newly constructed contigs were added to existing alignments [30], and all subsequent analyses were based on the merged alignments. Further sequence alignment and editing were carried out with BioLign Version 2.09.1 (Tom Hall, NC State Univ.) as described in [30]. New DNA sequences obtained for this study were deposited in GenBank under accession numbers GQ999668-GQ999777.

The cytoplasm genomes of *O. sativa *cultivars from independent domestication events have been used to distinguish cultivar groups [15,43,44]. We assessed the origins of cytoplasm genomes in weedy rice using one chloroplast [Orf100, [44]], and two mitochondrial [SSV500 and SSV39, [45]] markers in all 58 weedy rice accessions, and 82 *Oryza *samples from our panel and those from [30] for which DNA was available. These PCR-based markers amplify regions in the chloroplast or mitochondria containing large indels (69 bp to 500 bp), which can be visualized on a 1% agarose gel. Reaction conditions were as in [44] and [45]. We assumed maternal inheritance for cytoplasmic genomes, and combined the three markers into a single cytotype for analysis.

### Population structure

We assessed population structure using the Bayesian clustering program InStruct [46], which is similar to the commonly used STRUCTURE [47], but was developed specifically for identifying population structure in inbreeding species. Cultivated and weedy *Oryza *tend to self-fertilize, while wild *Oryza *outcross more frequently (10 to 60%) [20,26]. InStruct does not assume Hardy Weinberg equilibrium within populations, which can result in over-splitting in populations with a history of inbreeding [46,48]. We created genotype data from phased haplotypes inferred for each STS fragment using PHASE 2.1 [49].

We inferred population structure using two data sets: one included only U.S. weedy rice accessions (N = 58) and the second contained all individuals used in this study (N = 209). To determine the number of populations (*K*) that best approximates population structure, we tested a range of purposefully extreme *K*: *K *= 2 to 20 for the complete data set, and *K *= 2 to 15 for the weedy rice dataset. For each value of *K*, five replicates were carried out with an initial burn-in of 100,000 followed by 500,000 iterations using the "infer population structure and the individual selfing rates" option for final simulations. Sizes of burn-in and simulation number were found sufficient based on the Gelman-Rubin estimate of chain convergence for preliminary trial runs of various lengths (data not shown). All InStruct analyses were run on a computer cluster freely available at the Computational Biology Service Unit of Cornell University http://cbsuapps.tc.cornell.edu/InStruct.aspx. We used the Deviance Information Criterion (DIC) scores provided in the InStruct output to determine the number of populations that best fit our data. The *K *with the lowest average DIC score of the five replicates was considered to best describe population structure. For the model with the lowest mean DIC score, we checked for consistency in estimates of membership coefficients and split locations by estimating the correlation between ancestry membership matrices of replicate model runs with the R package simco [50]. InStruct results were plotted using R v2.6.2 [51].

### Summary statistics

Summary statistics for each STS locus and population of interest, including nucleotide diversity (θ_W _and θ_π_), Tajima's D, polymorphic loci (P), number of segregating sites (S), and population unique alleles/haplotypes were calculated as described in [30]. Site type determination was based on annotations of the *O. sativa *genome (TIGR v. 5 January, 2008).

Levels of population differentiation were estimated using F_st_, calculated after [52], using modifications of [53], which drops singleton SNPs. We calculated F_st _for each STS fragment by taking the mean F_st _of all SNPs per fragment, and then calculated the grand mean over all STS fragments, counting non-polymorphic fragments as zeros. Negative values of SNP F_st _were changed to zero before taking means of individual SNPs per STS fragment [53].

### Demographic models of weedy rice evolution

To infer the demographic history most consistent with the observed patterns of polymorphism in U.S. weedy rice, we used a full likelihood method, IMa (Isolation with Migration analytic; [54,55]), and an approximate Bayesian computation (ABC) method that relies on summary statistics [56].

A description of the demographic model and assumptions of the IMa analyses are provided in Additional File 4 and below we discuss details specific to our implementation. Three population pairs were considered, and each IMa analysis used only STS polymorphic within each population pair, as preliminary runs including invariant loci would not converge in a reasonable time. All pairs contained a similar number of polymorphic loci (27-32); thus exclusion of invariant loci does not preferentially affect parameter estimates in any group. We used a neutral mutation rate of 1 × 10^-8 ^[57], derived from synonymous site divergence at the maize *Adh *loci [58] to convert ML estimates to years and number of individuals. Both cultivated and weedy rice are, on average, annual plants under field conditions due to harvesting and cultivation practices [17], and we assumed a generation time of one year. Note that excluding monomorphic STS effectively increases the baseline mutation rate by ~1.6 (48/30), but this value is within error ranges of mutation range estimates, and does not affect scaling of parameters across groups. For all runs, we assumed that migration between populations was symmetrical, and set the maximum prior for population sizes to be equal. For final runs, we used a burn-in of 5,000,000 and recorded simulations for an additional 5,000,000 iterations using 10 chains and a two-step geometric heating scheme. To check for convergence, we ran each parameter set three times with a different starting random seed. IMa command lines were: ima -b 5000000 -l 50000 -m1 25 -m2 25 -f g -n 10 -g1 0.7 -g2 0.8 -p345 -q1 5 -k 3 -t 5 -s12307.

**Figure 2:**
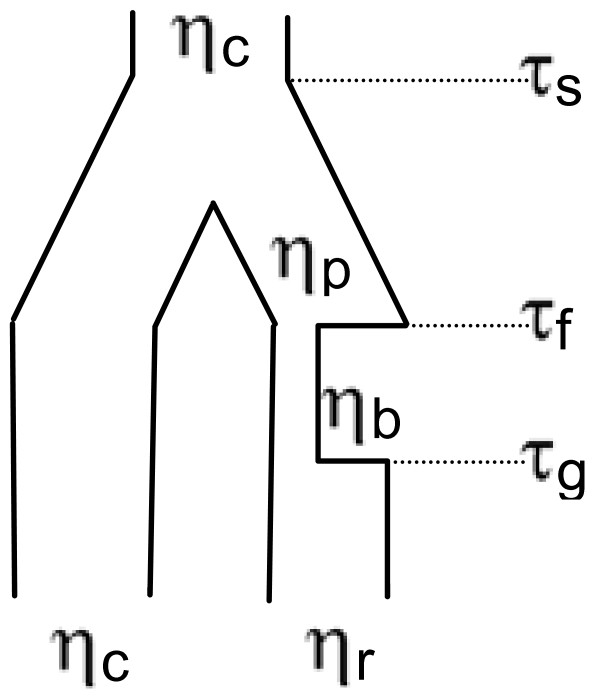
**Demographic model for ABC analysis**. This model assumes that the initial weedy rice founder (effective population size η_p_), split from an *Oryza *group (effective population size η_c_) in Asia at time τ_s _generations in the past. At time τ_f_, lineages from the founder population, η_p, _were introduced to the U.S. and experienced a bottleneck of size η_b _before recovering to current population size η_r_, instantaneously at time τ_g_.

The demographic model used in ABC analyses is shown in Figure 2, and is similar to models used to assess population divergence and crop domestication [e.g. [35,54,59,60]]. We used this model to test which scenario is most consistent with the demographic history of U.S. weedy rice: i) prior to introduction to the U.S., from a domesticated progenitor in Asia (~12,000 years before present [ybp]); ii) de novo, from cultivated germplasm introduced to the U.S.; and/or iii) from wild populations prior to domestication (Figure 2, Additional file 4) All simulations were performed using MS [61], and were conditioned on the population mutation rate θ = 4Nμ, where 4N is the reference population size and μ is the per nucleotide per generation mutation rate (μ = 1 × 10^-8^, as above). We used the observed mean silent site θ_w _for *O. rufipogon *from [30] to estimate θ = 4Nμ, as the allele frequency spectrum of *O. rufipogon *is consistent with a population evolving at a constant size. The population recombination rate, ρ, was assumed to be identical to θ, similar to other recent studies [30,62]. We considered weedy rice to be effectively entirely selfing and scaled timing parameters using 2N, rather than 4N.

We assumed that the population size of the progenitor of weedy rice has remained constant and set η_c _equal to η_p _for the duration of an individual simulation. Priors for η_c _were based on the ratio η_c_/4N and ranged from 0.1 to 0.7. These limits were based on the observed ratio of silent site θ_w, crop_/θ_w,*O. rufipogon*_. Priors for the current and bottleneck population size of the weed were based on the ratios η_r_/η_c_, and η_b_/η_r_, and ranged from 0 - 1 and 0-η_r _respectively. Priors for time of population expansion (τ_g_), founding in the U.S. (τ_f_), and time of divergence (τ_s_) were based on the known history of cultivated rice in the U.S. and timing of domestication. The upper limit for τ_g _was chosen to coincide with the rapid expansion of cultivated rice, which began around 1870 in the southern rice belt. Similarly, τ_f _was assumed to have occurred after cultivated rice was introduced into the U.S. and was constrained to be less than 400 years ago. Priors for τ_s _ranged from τ_f _to 50,000 ybp, and were chosen to be consistent with divergence occurring prior to domestication (τ_s _= 12,000-50,000), post domestication in Asia (τ_s _≤ 12,000), and at the time of founding in the U.S. (τ_s _= τ_f_). A grid of prior values for the three timing parameters and η_c _was generated, and the MS command line and further details on parameter ranges are given in Additional file 4.

Summary statistics and observed data were calculated using data pooled from all 48 STS fragments [after 30]. We chose summary statistics shown to be sensitive (correlated) to changes in population growth and timing of divergence [63]. These statistics also illustrate a key pattern observed in the data: that weedy rice groups contained a subset of genetic diversity present in putative ancestral populations (see results). We used eight statistics: θ_π _for both populations combined, the number of segregating, fixed, and private sites in weedy populations and their putative cultivated progenitors, and the number of shared sites between weeds and their putative progenitors. A similar set of summary statistics were used to infer demographic history in *Zea *[64].

We employed a similar rejection approach as in [35,65] and used the proportion of accepted simulations to calculate the approximate likelihood for a given demographic scenario. For each of the scenarios described above, we performed ~850,000 simulations. All processing and analysis of MS output were performed using R.

## Results

### Marker data

The 48 sequenced STS ranged in aligned length from 400 to 921 base pairs (bp) over all accessions, for a total of ~24,145 bp aligned sequence per accession. We observed 827 SNPs in our entire dataset. Thirty-three SNPs had more than two alleles, primarily (73%) due to alternative states present in the outgroup species (*O. barthii *or *O. meridionalis*). These SNPs were excluded from analyses when occurring in targeted groups. Insertions and deletions (indels) were not used in haplotype determination or calculation of summary statistics (unless segregating sites occurred within an indel, which was rare). Heterozygotes were observed almost exclusively in *O. rufipogon*, and only two weedy rice and four cultivated *O. sativa *accessions had heterozygous sites.

Except for one weedy rice accession, the three-cytoplasm markers amplified in all individuals screened (n = 139). We observed the same sized length variants (i.e. the size of deletion in base pairs) for each marker that were found in [47,48] (Table 1). In general, we found that the cytotypes had similar distributions within cultivated *O. sativa *varieties as reported in [44] and [43] (Table 1, Additional files 1 and 2). However, unlike [45], we did not find complete linkage between the mitochondrial markers.

**Table 1:** Cytoype frequency in weedy rice populations and potential sources in *Oryza*

	Cytotype*					
	
Population	N.N.D	N.N.N	D.N.N	N.D.D	D.D.D	D.D.N
BHA1	0.60	.	.	0.20	0.13	0.07
BHA2	0.71	.	.	0.29	.	.
BRH	.	.	.	.	.	1
SH	.	.	.	.	.	1
*aus*	.	.	0.50	0.17	.	0.33
*indica*	0.20	.	.	0.20	.	0.60
*tropical japonica*	0.63	0.13	.	0.13	.	0.13
U.S. cultivars	1	.	.	.	.	.
*O. rufipogon*	0.07	.	.	0.37	0.03	0.53

* Data from three markers combined to generate single cytotype in the following order: cpDNA Orf100, mtDNA SSV500, and mtDNA SSV39. Length variants were scored as N, indicating non-deletion, and D, indicating deletion; D.D.N is reported to be most common cytotype in *indica *and N.N.D in *japonica *[47].

### U.S. weedy rice population structure

**Figure 3:**
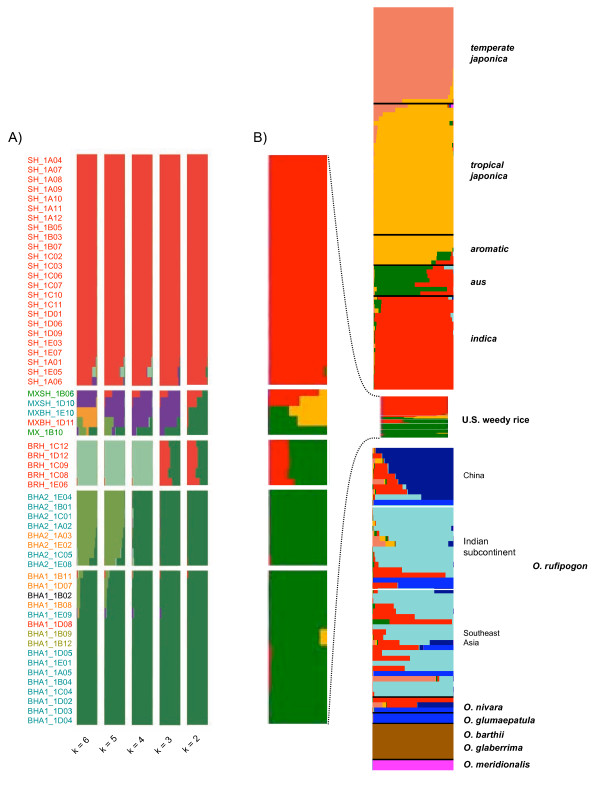
**Population structure assessed by InStruct**. Each individual accession is represented by a colored bar, partitioned to reflect an individual's relative proportion of genetic membership in a given cluster *K*. A) Results of analysis for U.S. weedy rice accessions (n = 58), showing progression of clustering as *K *increased to optimal *K *= 6. The order of accessions is the same as in Additional file 1 and in all plots. B) Population structure results for the entire *Oryza *panel (n = 209), showing clustering at optimal *K *= 9. Inset plot shows clustering of U.S. weedy rice. Samples are in the same order as Additional file 2, and *O. rufipogon *samples are sorted by country of origin. Weedy rice accession names color coded by cytotype: DDD light green, NDD orange, NND blue, DDN red, NNN dark green, Black - no data

To determine the number of weedy rice populations occurring within the U.S., we used InStruct and a data set containing only weed accessions (Additional file 1; n = 58). Based on DIC scores (Additional file 5), we found that population structure is most consistent with a model containing six groups (*K *= 6) (Figure 3A). Individuals belonging to the same cluster tend to have similar grain morphologies. At *K *= 2, individuals with straw hulls that lack awns (SH = straw hull) are differentiated from other hull phenotypes. With increasing *K*, SH individuals remained in a single cluster, while the non-SH group was further subdivided into five subpopulations (Figure 3A). Based on the predominant grain phenotype (i.e. hull color and presence or absence of an awn) in each population, we designated these as: BHA1 (black hull awned 1), BHA2 (black hull awned 2), BRH (brown hull awned), MXSH (mixed straw hull), and MXBH (mixed black hull awned). With one exception, all 24 weedy rice individuals with straw-colored hulls and no awns in our panel clustered in the SH population. All other clusters, however, contained multiple grain phenotypes (Additional file 1). For example, ~73% of individuals in BHA1 and ~63% of BHA2 had awns and a black hull, and ~60% of BRH individuals had brown hulls and awns. Similar results were obtained when analyses were run with STRUCTURE (data not shown).

### *Oryza *population structure

To identify potential source(s) for U.S. weedy rice within *Oryza*, we used InStruct and a dataset that included all accessions in our panel (n = 209). The best fitting model contained nine populations (*K *= 9) (Figure 3B, Additional files 2 and 5). Cluster membership was generally consistent with previous research [30,66]. InStruct identified *O. sativa *varieties *aus*, *indica*, *tropical japonica *and *temperate japonica *as distinct populations; however, our dataset lacked resolution to differentiate *tropical japonica *and *aromatic *accessions. The fourteen U.S. cultivars included in this study clustered with *tropical japonica*, as expected, and historic and modern cultivars were not differentiated.

Approximately four clusters were observed within the wild ancestor of cultivated rice, *O. rufipogon *although most individuals appeared to be admixtures (Figure 3B). Many *O. rufipogon *individuals shared some ancestry with *indica*, but only five had membership coefficients greater than 50%. None of these were indicated as hybrids in the passport data available, and admixture may be due to shared ancestry, rather than recent hybridization. Consistent with previous research [20,67,68] no distinct *O. nivara *cluster separate from *O. rufipogon *was observed. African cultivated rice, *O. glaberrima*, and its progenitor, *O. barthii*, formed a distinct cluster, as did the two *O. meridionalis *samples. *O. glumaepatula *samples, on the other hand, clustered with three *O. rufipogon *and one *O. nivara *(Figure 3B, Additional file 2).

### Origins of U.S. weedy rice populations

To determine the putative progenitors of U.S. weedy rice, we used the results of the two InStruct analyses, combined with the genotyping results for the three-cytoplasm markers. All of the SH individuals identified by InStruct (Figure 3A) cluster with *indica *when all samples are used (Figure 3B). All SH accessions had the same cytotype, which was also the most frequent in *indica *(60%) and *O. rufipogon *(53%) (Table 1), and was found in all of the *O. rufipogon *and *O. nivara *accessions that shared greater than 50% membership with *indica *(Additional file 2).

Both black hulled weedy rice groups, BHA1 and BHA2, cluster primarily with *aus *and are not differentiated in the InStruct analysis that included all individuals (Figure 3B). Interestingly, the most frequent BHA1 (60%) and BHA2 (71%) cytotype did not occur in our *aus *sample, but is most common in *tropical japonica *(63%), and rare in *indica *(20%) and *O. rufipogon *(7%) (Table 1). However, two other cytotypes found in BHA1 and BHA2 were also found at high frequency in *aus*. Two BHA1 individuals and an *O. rufipogon *accession from India shared a cytotype that was absent in all other accessions (Additional file 2).

The InStruct analyses suggest that the BRH population is either the result of hybridization between *indica *and *aus*, the SH and BHA weedy groups, *indica *and BHA, or *aus *and SH (Figure 3A and 3B). The BRH group contained a subset of the diversity found in the SH and BHA groups (10 of the most frequent STS haplotypes [MFH] in BRH were exclusive to BHA1 and BHA2, and six to SH; the remaining 32 were common to all weedy populations) consistent with hybridization among weedy groups in the U.S. All BRH individuals have the same cytotype as SH weeds, suggesting a maternal SH lineage. No heterozygotes were observed, which would be expected from early generation hybrids; however, heterozygosity may have been affected by selfing at the USDA stock center.

InStruct results also indicate that hybridization between *tropical japonica *varieties grown in the U.S. and weedy rice has occurred. Population MXSH contains two individuals that share genetic membership with both *indica*/SH and *tropical japonica *(Figure 3B). The MXSH population is also notable in that weedy rice is likely the paternal rather than maternal parent, as observed cytotypes are absent from SH weeds, but occur in *tropical japonica *(Additional files 1 and 2, Table 1). Individuals in the MXBH group were identified as admixtures between *aus*/BHA and *tropical japonica *(Figure 3B). Both accessions in MXBH have the same cytotype (Additional file 1), which is absent in *aus*, but found in BHA groups and *tropical japonica*. Outside of the MXSH and MXBH populations, only one accession shared membership with *tropical japonica *(Figure 3B).

Three of the five putative hybrids we identified were listed as suspected crosses based on morphological observations made at the time of collection (Additional file 1). Three modern U.S. cultivars (M202, Bengal, and Palmyra) appear as admixtures of *temperate *and *tropical japonica *in our analyses, in agreement with known pedigree data. This suggests our data is sufficient for identifying relatively advanced generation hybrids and supports our designation of weedy hybrids.

### Genetic diversity in weedy rice

**Figure 4:**
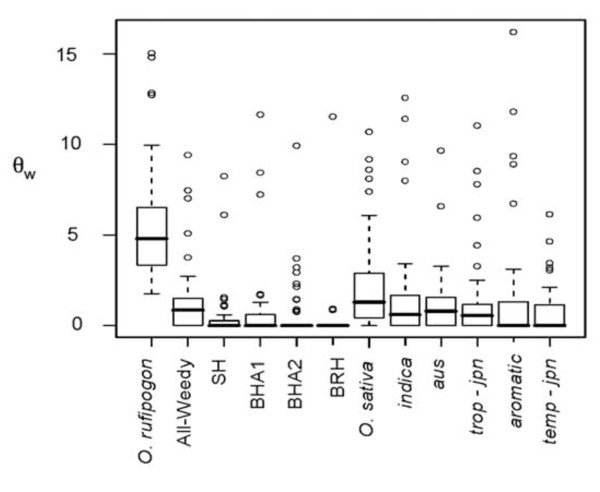
**Silent site θ_w _within *Oryza *populations**. Boxplot of θ_w _calculated per kb, where boxes show interquartile ranges and median. The whiskers extend to 1.5 times the interquartile range and outliers are shown as open circles.

Genetic diversity statistics were calculated for all 48 STS for U.S. weedy rice groups and potential sources within *Oryza *(Table 2, Additional file 3). For all measures of genetic diversity, weedy rice, considered as a single group, was genetically depauperate, with less than ~40% and ~70% of the diversity present in *O. sativa *and *O. rufipogon *respectively. Mean θ_W _and θ_π _are highest in *O. rufipogon*, intermediate for *O. sativa *groups and lowest in U.S. weedy rice. We also found that the distribution of nucleotide diversity is heterogeneous across loci for all groups, but particularly in weedy and cultivated groups where a few loci are atypically polymorphic (Figure 4, Additional file 3).

**Table 2:** Mean diversity measures for 48 STS loci

		U.S. weedy rice populations						*O. sativa *populations					
		
Statistic	Site type	*O. rufipogon*	All Weedy	SH	BHA1	BHA2	BRH	*O. sativa*	*indica*	*Aus*	***temp***. *japonica*	***trop***. *japonica*	*aromatic*
θ_*W *_per Kb	All	5.606	1.363	0.490	0.827	0.619	0.278	2.292	1.650	1.092	0.814	1.370	1.590
	Silent	7.787	1.694	0.638	0.933	0.695	0.349	2.985	2.216	1.401	1.050	1.694	2.353
	Non-synonymous	2.830	0.540	0.300	0.420	0.460	0.100	1.030	0.670	0.950	0.300	0.570	0.340
	Synonymous	9.015	1.896	0.585	0.736	0.424	-	3.204	2.225	1.308	0.263	1.716	3.510
													
θ_π _per Kb	All	4.382	1.480	0.564	0.750	0.564	0.230	2.461	1.614	1.189	0.552	1.073	1.447
	Silent	6.354	1.848	0.692	0.829	0.657	0.311	3.278	2.179	1.568	0.650	1.337	2.176
	Non-synonymous	1.790	0.754	0.337	0.463	0.373	0.080	0.750	0.584	0.883	0.360	0.405	0.300
	Synonymous	7.019	1.312	0.505	0.650	0.476	-	4.228	1.982	1.026	0.345	1.257	3.029
													
Tajima's D	All	-0.729	0.220	-0.441	-0.177	0.042	-0.266	0.232	-0.026	0.092	-0.754	-0.773	-0.408
	Silent	-0.607	0.240	-0.404	-0.173	0.017	0.013	0.354	0.039	0.145	-1.043	-0.731	-0.451
	Non-synonymous	-0.724	0.358	-0.155	-0.132	-0.185	-0.197	0.339	-0.132	0.277	-0.816	-0.685	-0.214
	Synonymous	-0.442	-0.343	-0.355	-0.165	0.484	-	0.383	0.030	1.081	-0.498	-0.826	-0.629
													
# of polymorphic STS (all sites)		48	35	12	14	12	3	43	33	26	21	33	19
# of polymorphic STS (silent sites)		48	33	11	13	11	2	39	30	23	18	26	18

In general, U.S. weedy rice groups contain a subset of diversity observed in their most closely related cultivated *O. sativa *populations. SH weedy rice contains only ~30% of the silent site variation found in *indica*, while the BHA1 and BHA2 groups harbor between 50-67% of the variation found in *aus*. For a majority of the STS fragments, weedy rice groups and putative progenitor shared the same MFH (83% of STS fragments in *indica *and 73% of STS fragments in *aus*). We did not observe high frequency or specific haplotypes that would suggest weedy rice is a product of recent hybridization with *O. rufipogon*.

We estimated differentiation between U.S. weedy rice populations and their closely related *Oryza *groups using mean and median values of STS F_st _(Table 3). SH and BHA1 were more diverged than *indica *from *aus*, and the most highly differentiated population pair tested (Table 3). Populations BHA1 and BHA2 were not greatly differentiated but both were more diverged from *aus *than SH from *indica*, consistent with a higher number of private SNPs and presence of one fixed SNP in BHA/*aus *comparisons (Additional file 6). Low median F_st _values between weedy populations and their closest *Oryza *relatives show that estimates of population differentiation are driven by a few loci (Additional file 6).

**Table 3:** Mean and median STS F_*st *_between U.S. weedy populations and putative *Oryza *progenitors

Population	*indica*	SH	*aus*	BHA1	BHA2	*O. rufipogon*
*indica*		0.050^b^ (0 - 0.56)	0.013 (0 - 0.83)	0.240 (0 - 1)	0 (0 - 1)	0.036 (0 - 0.423)
SH	0.132^a^		0 (0 - 0.98)	0 (0 - 1)	0 (0 - 1)	0.041 (0 - 0.428)
*aus*	0.170	0.290		0 (0 - 1)	0 (0 -1)	0 (0 - 0.217)
BHA1	0.285	0.304	0.198		0 (0 - 1)	0.070 (0 - 0.407)
BHA2	0.241	0.239	0.162	0.090		0.070 (0 - 0.407)
*O. rufipogon*	0.0580	0.049	0.034	0.078	0.070	

^a ^Mean STS F_*st *_among population pairs

^b ^Median STS F_st _among population pairs with ranges in parenthesis

### Estimates of demographic parameters

We used IMa and ABC to infer time of divergence and population sizes for the two main weedy rice groups (SH and BHA1) and their closest *Oryza *relatives. The results of a single simulation for demographic parameters from IMa analyses are shown in Table 4 and Additional file 7, and posterior probability density curves for parameter estimates are shown in Additional file 8. For all population pair comparisons, differences in parameter estimates among the three simulation runs were small (less than 5%) and the 90% posterior density intervals (HPD) overlapped, suggesting chain convergence. The maximum likelihood (ML) estimates of current and ancestral effective population size (N_e_) were consistent with expectations that U.S. weedy rice populations have experienced population bottlenecks (Table 4, Additional file 7). ML estimates of N_e _for BHA1 (~2,472 individuals) and SH (~1,000 individuals) are an order of magnitude smaller than for their ancestral populations (77,148 and 74,397 respectively). ML estimates of current weedy rice N_e _were also smaller than estimates of current N_e _for their relatives. N_e _estimates for *aus *and *indica *across simulations were similar, with HPD intervals covering similar ranges in population size (Table 4, Additional file 7). The larger estimates for *indica *are consistent with the limited geographical distribution of *aus*. To account for high rate of selfing in cultivated rice, our analysis is based on a single haplotype (i.e. chromosome) per individual, and, therefore, depending on the actual degree of selfing (exact values are unknown), population size estimates may be, at most, twice as large [69].

**Table 4:** Rescaled ML estimates and 90% posterior density intervals (HPD) of demographic parameters for three population pairs

Population Pair	*N*_*e1 *_^*a*^	*N*_*e2 *_^*a*^	*N*_*eA *_^*b*^	*t* ^*c*^
BHA1-*aus*^d^	2,472 (1,483 - 4,450)	5,439 (2,472 - 12,854)	77,148 (4,3013 - 166,613)	9,939 (3,975 - 27,829)
SH-*indica*^d^	1,000 (299 - 1,897)	15,978 (9,487 - 30,458)	74,397 (36,548 - 244,662*)	31,955 (9,986 -81,887)
*aus-indica *^d^	4,536 (2,016 - 11,592)	13,102 (4,535 -19,653)	43,341 (20,157 -246,922)	6,047 (605 - 241,880)

^a ^Effective population size (N_e_) in order listed (i.e. BHA1 is N_e1 _and *aus *is N_e2_) reported as number of individuals; numbers in parenthesis represent the 90% posterior density intervals.

^b ^Ancestral population effective population size; numbers in parenthesis represent the 90% posterior density intervals.

^c ^Population split times in years. Numbers in parenthesis represent 90% posterior density intervals. Asterisks indicate posterior density intervals that exceed maximum priors for the parameter.

^d ^Number of loci used in analysis: BHA1-*aus *27; SH-*indica *28; *aus-indica *32

IMa-based estimate of divergence time between *aus *and *indica *was ~6,047 ybp, (Table 4, Additional file 6), with a wide HPD interval (605 to 241,880 ybp). Divergence time estimates for SH from *indica *(~31,995 ybp) and BHA1 from *aus *(~9,939 ybp) predate the introduction of cultivated rice to the U.S. (~1690's), and its establishment in the southern rice belt (>150 years). However, confidence HPD intervals for all estimates are very large and overlap (Table 4, Additional file 7).

Obtaining estimates of migration between populations from our IMa runs was problematic. Initial runs of models that did not include migration, under the assumption that gene flow between weeds in the U.S. and cultivars in Asia is unlikely, did not converge. Including migration improved estimates for remaining parameters. However, for all population pairs, estimates of migration are not reliable, as posterior distributions did not converge within prior ranges (Additional file 8), suggesting that, under short evolutionary time scales, with this dataset, IMa may confound recent divergence with ongoing gene flow.

**Figure 5:**
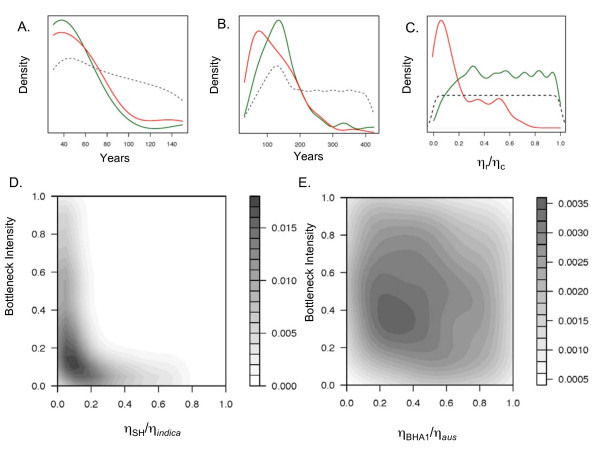
**Results of the ABC analysis**. Priors are plotted as a dashed black line A) time of population expansion: τ_g SH _in red and τ_g BHA1 _in green, B) time of founding in U.S. τ_f SH _in red and τ_f BHA1 _in green, C) η_SH_/η_*indica *_in red, η_BHA1_/η_*aus *_in green. Contour plots of the approximate likelihood for current weedy population size as a function of bottleneck intensity, which is the percent decline in population size during the bottleneck, D) SH, and E) BHA1.

To further explore the demographic history of U.S. weedy rice, we carried out coalescent simulations under four demographic scenarios, and compared obtained summary statistics with those of the observed data (Table 5, Figure 5). For both weedy rice groups, divergence from cultivated relatives prior to domestication was not supported (Table 5). However, divergence prior to arrival to the U.S. was supported for BHA1 from *aus *(Table 5). For SH weedy rice, recent divergence from *indica*, occurring either in Asia or in the U.S. was found to be most likely (Table 5). Similar to our IMa analyses, we found that population bottlenecks have played a role in the evolution of U.S. weedy rice, but bottleneck intensity appears to have impacted SH more than BHA1 (Figure 5C, D, and 5E). For both weedy populations, the distribution of τ_g _indicates recent and simultaneous population expansion (Figure 5A), consistent with the known history of expansion of cultivated rice production in the past 100 years in the Southern rice belt. Founding in the U.S. for both BHA1 and SH appears to have occurred within the past 200 years (Figure 5B).

**Table 5:** Approximate likelihoods for divergence scenarios

	Timing of divergence			
	
Population Pair	Introduction to U.S.^b^	Post domestication^c^	Domestication^d^	Prior to domestication^e^
BHA1 - *aus*	0.005	0.098	0.061	0.02
SH - *indica*	0.061	0.022	0.001	0

^a ^The approximate likelihood is shown, calculated out of total number of simulations for each scenario.

^b ^τ_s _= τ_f _and ranges from 30 to 400 years.

^c ^τ_f _= 1000 - 7000

^d ^τ_f _= 7000 - 12000

^e ^τ_f _= 12000 - 50000

## Discussion

### The evolutionary origins of U.S. weedy rice

Current weedy rice populations in the U.S. are morphologically diverse, and we find that population structure in weedy rice is correlated with hull morphology. The two major weedy rice groups occurring in the U.S. are most closely related to the exotic cultivated rice varieties, *aus *and *indica*. Our data thus provides strong evidence that weedy con-generics can evolve directly from domesticated backgrounds, a result that has been little reported/confirmed to date [5,9,18].

Similar to previous morphometric and molecular marker studies [13,14,16], weedy rice individuals that have straw hulls and no awns (SH) cluster primarily with *O. sativa indica *(Figure 3). Other hull morphologies, including black and straw hull with awns (BHA1, BHA2), cluster primarily with *O. sativa aus *(Figure 3), a relationship also recently detected with microsatellites [13]. Unlike previous microsatellite based studies [13,14], we did not find conclusive evidence for contribution of wild *Oryza *species to U.S. weedy rice. Although some *O. rufipogon *and *O. nivara *accessions clustered with *indica *and SH weedy rice (Figure 3), only two out of 51 accessions had the same level of shared genetic membership (>80%) with SH weedy rice as all *indica *accessions. Moreover, accessions of *O. rufipogon *and *O. nivara *that clustered with SH groups in our analysis do not share hull morphology or any unique alleles with weedy accessions, unlike *indica*, supporting shared ancestry as the most likely explanation for the clustering pattern. Black hulls and awns are a common phenotype in *O. rufipogon *and *O. nivara*, but no accessions from this group clustered with BHA weedy rice. *Aus *cultivars, however, often have dark hulls and awns. The results of our clustering analyses combined with morphological data suggest that the main U.S. weedy rice groups evolved primarily from *aus *and *indica *genetic backgrounds.

Although clustering of weedy rice groups with cultivated relatives could also be due to common descent from a shared ancestral founding gene pool, the pattern of shared polymorphisms among weedy and cultivated groups is more consistent with direct descent from domesticated ancestors. Most of the SNPs found in the SH and BHA groups are a subset of those found in *indica *and *aus*, respectively (Additional file 6). This is particularly striking for the SH group, which contains only one non-singleton SNP not also found in *indica*, fewer than what it has with respect to *O. rufipogon *(Additional file 6). Moreover, in each main weedy rice group (SH and BHA1), the most frequent haplotype (MFH) at each STS locus was most often the MFH observed in its putative progenitor group (data not shown). The greater divergence and number of private SNPs seen in the BHA groups with respect to *aus*, as well as differences in some cytotypes, however, suggest that demographic histories (e.g. magnitude of bottleneck, founding events, time of introduction) differ between the BHA and SH groups.

The close relationship of weedy rice with cultivated groups not grown in the U.S. suggests that both major weed groups were introduced either as stock seed contaminants or escaped breeding material. Although the majority of rice grown commercially in the U.S is *tropical japonica *[27,39], extensive opportunities for the intentional and unintentional introduction of *Oryza *germplasm have occurred. During the establishment of rice industry in the southern rice belt (~1860-1900), rice germplasm collected by the USDA was given to farmers directly for testing [70], potentially facilitating the spread and escape of weedy rice. During this time, farmers also commonly purchased seed from outside the U.S., which likely included representatives of all major *O. sativa *varieties.

### The timing of weed evolution

If U.S. weedy rice groups originated from cultivated ancestors, it is of interest to determine whether divergence of the weeds occurred prior to or concurrent with their introduction to the U.S., and how divergence is related to the timing of domestication. We first estimated divergence time between *aus *and *indica *cultivar groups, which likely stem from the same domestication event. The ML estimate of ~6,000 years (Table 4) is reasonable, given that the commonly accepted time for domestication is ~10,000 years ago; however, confidence intervals for the estimate are large, consistent with the difficulty in estimating population parameters for very recent events [71]. In contrast, IMa estimates for divergence of weedy groups from their cultivated relatives were surprisingly ancient, although, again, confidence intervals were very large. ABC coalescent simulations, on the other hand, supported a very recent SH-*indica *divergence, within the past 100 years, but divergence for BHA1 and *aus *occurring within the past 7,000 years (Table 5).

We considered two possible explanations to account for the discrepancy between SH-*indica *divergence time estimates obtained in each of our analyses. First, contribution of other groups to the weedy rice gene pool or unsampled variation in the putative progenitor could violate IMa assumptions that gene flow occurs only between population pairs, inflating estimates of divergence time. However, SH weedy rice contains a subset of the nuclear and cytoplasmic genetic diversity in *indica *(Table 2, Additional file 6); the only non-singleton private SNP in SH occurs at low frequency (13%), and was not found in any other *Oryza *group other than BHA weeds. Thus, introgression or incomplete sampling of *indica *diversity is an unlikely explanation of divergence estimates.

Alternatively, IMa divergence time estimates may be affected by the combination of an extremely strong bottleneck coupled with very recent divergence between *indica *and SH. Although, the IMa model is particularly suited to recently separated populations that are not under equilibrium [54], simulation studies to test sensitivity of IMa to extremely recent splits with no accumulation of divergent mutations have not been done (J. Hey, personal comm.). Both demographic analyses and the low levels of observed polymorphism support a very strong bottleneck for SH weedy rice (Tables 2 and 4, Figure 5). Since few lineages seem to have founded this weedy group, the divergence times obtained may represent the coalescence of these founders with the entire *indica *gene pool, and not the more recent split between weedy rice and progenitor. Observed patterns of polymorphism support the more recent divergence time estimated by ABC: SH either diverged from *indica *concurrent with its establishment in the U.S. (maximum of 400 ybp), or within 1000 ybp (Table 5).

Both demographic analyses suggested an older divergence of BHA1 from its putative *aus *progenitor, either after domestication, or close to the timing of domestication (Tables 4 and 5). In addition to one fixed site, the BHA1 group contains some private SNPs and cytotypes not observed in our *aus *sample (Table 2, Additional file 6). These patterns of polymorphism may indicate introgression of other *Oryza*, or incomplete sampling of *aus *diversity, which could have an effect on estimates of divergence time. Nuclear SNPs observed in BHA1 but not *aus *occurred at moderate frequencies (average 52%) and were also relatively frequent in other groups such as *O. rufipogon*, *O. nivara*, and *tropical japonica*, supporting the possibility of introgression. However, our Instruct analysis did not detect contribution of other *Oryza *groups to BHA weedy rice, and we have no a priori reason to believe our *aus *sampling did not capture the genetic diversity present in this geographically limited group. Given the shared ancestry of all cultivars, weeds, and *O. rufipogon*, BHA1 private alleles shared with other groups could be a result of lineage sorting since divergence from *aus*. Interestingly, the single fixed SNP differentiating BHA groups from *aus *was not observed in any other *Oryza *group, supporting longer divergence between BHA weedy rice and its putative progenitor. Our estimates suggest that the founders of the BHA1 weedy rice group split from their cultivated relatives several thousand years ago and therefore may have existed as weeds prior to their introduction to the U.S. The ABC analysis marginally supported the introduction of BHA weeds before SH, which is contrary to expected based on historical records; black hull awned plants were not recorded until the 1920's, and anecdotal evidence attributes their origin to a cultivar introduced to Louisiana and abandoned due to excessive shattering [17].

### The role of hybridization in U.S. weedy rice

In addition to multiple introductions, our results suggest that hybridization and introgression occurring post-founding have contributed to the development of morphological diversity in weedy rice populations. The BRH population is most probably a product of hybridization occurring in the U.S. between SH and BHA weedy rice (Figure 3). No *indica *or *aus *are grown in the U.S., and therefore, an additional introduction of a weedy or cultivated group to the country would be required if BRH were the result of hybridization between *indica-aus*, *indica-*BHA, or SH-*aus*. The high estimates of F_st _between SH and BHA1 (~0.32) indicates that gene flow is relatively infrequent between weedy groups. Prior research has suggested non-overlapping flowering time, high selfing rates, and height differences as possible mechanisms restricting gene flow between straw-hull and black-hull awned weedy types [16].

Evidence that *tropical japonica *cultivars grown in the U.S. have contributed to genomic backgrounds of weeds in our sample set is limited to a few individuals in the MX populations (Figure 3, Additional file 1). Several studies have observed both pre- and post-zygotic reproductive isolating barriers in experimental crosses between *tropical japonica *and weedy rice [16,25]. The existence of some barrier to gene flow is supported by the lack of more extensive hybridization in our sample. However, the barrier is "leaky," as both BHA and SH-*tropical japonica *hybrids are found (Figure 3B). Additionally, the maternal lineage of at least one hybrid was consistent with weedy rice being the paternal parent, and therefore, gene flow from the weed to the crop could be an alternative pathway for weed evolution. Although infrequent, the fact that hybridization occurs at all presents a challenge to the management and continued use of cultivars containing traits suspected to increase weed fitness, such as herbicide resistance.

## Conclusions

Our characterization of genome-wide patterns of SNP variation in U.S. weedy rice demonstrate that multiple introductions, bottlenecks, and hybridization among introduced lineages have been important in the evolution of weedy rice, and that different evolutionary histories can lead to similar weedy lifestyles. Contrary to previous studies, we do not find evidence that wild *Oryza *contributed directly to the genetic background of U.S. weedy rice groups. Together these results provide strong evidence that agricultural weeds can evolve directly from domesticated backgrounds despite experiencing significant bottlenecks and loss of genetic diversity.

The absence of any *tropical japonica *weedy types in the U.S. is puzzling, as these cultivars are considered better adapted to the temperate conditions of the Southern U.S. than *indica *and *aus *cultivars. Based on typical descriptions of *aus *and *indica*, it would seem that increased tolerance to cold, high dormancy, easy shattering, and lack of photoperiod sensitivity (though this trait is found in *aus*) may have evolved in U.S. weedy rice populations. It will be interesting to determine whether trait evolution supports pre-existence of the groups as weeds in Asia, or evolution of weediness upon introduction to the U.S. agroecosystem.

## Authors' contributions

ALC, KMO, and YJ conceived the experiments. MR, and CST collected the data. BLG contributed materials. MR analyzed the data. MR and ALC wrote the paper. All authors read, provided editorial assistance, and approved the final manuscript.

## Supplementary Material

Additional file 1 **Supplementary Table 1**. U.S. weedy rice accessions used in study. Click here for file

Additional file 2 **Supplementary Table 2**. *Oryza *accession information and coefficients of ancestry inferred by InStruct. Click here for file

Additional file 3 **Supplementary Table 3**. Information on STS fragments, primers, and diversity data. Click here for file

Additional file 4 **Supplementary text 1**. MS command line and description of parameter values. Click here for file

Additional file 5 **Supplementary Table 4**. Mean DIC (5 simulations) for InStruct analysis of A) only U.S. weedy rice (n = 58), B) all *Oryza *accessions (n = 209). Click here for file

Additional file 6 **Supplementary Table 5**. Number of segregating sites, fixed and shared polymorphisms and private sites between various *Oryza *groups. Click here for file

Additional file 7 **Supplementary Table 6**. ML estimates and 90% posterior density intervals (HPD) of demographic parameters for three population pairs. Click here for file

Additional file 8 **Supplementary Figure 1**. Marginal posterior probability curves for demographic parameters for each population pair comparison as estimated by IMa. All estimates have not been converted to individuals or years A) ancestral population size for *aus *- *indica*, B) time of divergence *aus - indica*, C) migration from *aus *to *indica *in red, migration from *indica *to *aus *in black, D) population size of *aus *in red and *indica *in black, E) ancestral population size for *aus *- BHA1, F) time of divergence *aus - *BHA1, G) migration from *aus *to BHA1 in red, migration from BHA1 to *aus *in black, H) population size of *aus *in red and BHA1 in black, I) ancestral population size for *indica *- SH, J) time of divergence *indica - *SH, K) migration from *indica *to SH in red, migration from SH to *indica *in black, L) population size of *indica *in red and SH in black. Click here for file
