# Supplementary material for: Genomic patterns of nucleotide diversity in divergent populations of U.S. weedy rice
Source: BMC Evol Biol. 2010 Jun 15;10:180. doi: 10.1186/1471-2148-10-180 (PMC2898691; doi:10.1186/1471-2148-10-180)
Supplement: Additional file 1 — Supplementary Table 1. U.S. weedy rice accessions used in study. [file 1471-2148-10-180-S1.DOC]

Supplementary Table 1. U.S. weedy rice accessions used in study

| **Study ID** | **USDA ID ^a^** | **Pop. Id ^b^** | **State** | **Hull** | **Awn** | **Cytotype^c^** | **Percent cluster ancestry ^d^** | | | | |
| --- | --- | --- | --- | --- | --- | --- | --- | --- | --- | --- | --- |
|  |  |  |  |  |  |  | **SH** | **BHA1** | **BHA2** | **BRH** | **Mix** |
| SH_1A04 | **1091-01** | SH | AR | Straw | No | DDN | 98 | - | - | - | - |
| SH_1A07 | **1098-01** | SH | MO | Straw | No | DDN | 98 | - | - | - | - |
| SH_1A08 | **1134-01** | SH | AR | Straw | No | DDN | 98 | - | - | - | - |
| SH_1A09 | **1135-01** | SH | AR | Straw | No | DDN | 98 | - | - | - | - |
| SH_1A10 | **1141-01** | SH | AR | Straw | No | DDN | 98 | - | - | - | - |
| SH_1A11 | **1160-01** | SH | AR | Straw | No | DDN | 98 | - | - | - | - |
| SH_1A12 | **1179-01** | SH | LA | Straw | No | DDN | 98 | - | - | - | - |
| SH_1B05 | 1995-15 | SH | AR | Straw | No | DDN | 98 | - | - | - | - |
| SH_1B03 | 16B | SH | AR | Straw | No | DDN | 98 | - | - | - | - |
| SH_1B07 | 1996-05 | SH | MS | Straw | No | DDN | 98 | - | - | - | - |
| SH_1C02 | **1001-01** | SH | AR | Straw | No | DDN | 98 | - | - | - | - |
| SH_1C03 | **1002-02** | SH | AR | Straw | No | DDN | 98 | - | - | - | - |
| SH_1C06 | **1047-01** | SH | LA | Straw | No | DDN | 98 | - | - | - | - |
| SH_1C07 | **1073-02** | SH | MO | Straw | No | DDN | 98 | - | - | - | - |
| SH_1C10 | **1190-01** | SH | LA | Straw | No | DDN | 98 | - | - | - | - |
| SH_1C11 | **1199-01** | SH | MO | Straw | No | DDN | 98 | - | - | - | - |
| SH_1D01 | **1344-02** | SH | MO | Straw | No | DDN | 98 | - | - | - | - |
| SH_1D06 | 1995-12 | SH | LA | Straw | No | DDN | 98 | - | - | - | - |
| SH_1D09 | 1996-08 | SH | MS | Straw | No | DDN | 98 | - | - | - | - |
| SH_1E03 | **1210-02** | SH | MO | Straw | No | DDN | 98 | - | - | - | - |
| SH_1E07 | **1333-02** | SH | MO | Straw | No | DDN | 98 | - | - | - | - |
| SH_1A01 | **1004-01** | SH | MO | Straw | No | DDN | 91 | 1 | 2 | 4 | 3 |
| SH_1E05 | **1163-01** | SH | LA | Straw | No | DDN | 74 | 2 | 2 | 17 | 5 |
| SH_1A06 | **1196-01** | SH | AR | Straw | No | DDN | 74 | - | - | 1 | 24 |
| MXSH_1B06 | 1996-01 | MXSH | AR | Straw | No | NNN | 3 | - | - | - | 96 |
| MXSH_1D10 | 2002-51 | MXSH | AR | Straw | Short | NND | - | - | - | - | 97 |
| MXBH_1E10 | **2002-2-p21** | MXBH | AR | Black | No | NND | - | - | - | - | 96 |
| MXBH_1D11 | 2004-1-A | MXBH | AR | Black | Yes | NND | - | 1 | 28 | 2 | 69 |
| MX_1B10 | MS4R788_93 | MX | MS | Straw | Yes | NNN | - | 42 | 37 | 2 | 19 |
| BRH_1C12 | **1300-02** | BRH | MO | Brown | Short | DDN | - | - | - | 98 | - |
| BRH_1D12 | **1183-01** | BRH | AR | Straw | Yes | DDN | - | - | - | 98 | - |
| BRH_1C09 | **1111-01** | BRH | AR | Straw | Yes | DDN | - | - | - | 98 | - |
| BRH_1C08 | **1092-02** | BRH | MS | Brown | Yes | DDN | - | 1 | 1 | 97 | - |
| BRH_1E06 | **1120-02** | BRH | MO | Brown | Yes | DDN | 4 | - | - | 96 | - |
| BHA2_1E04 | **1214-02** | BHA2 | LA | Black | Yes | NND | - | 2 | 97 | - | - |
| BHA2_1B01 | **1188-01** | BHA2 | MS | Black | Yes | NND | - | 2 | 97 | - | - |
| BHA2_1C01 | TX4 | BHA2 | TX | Black | Yes | NND | - | 2 | 96 | 2 | - |
| BHA2_1A02 | **1025-01** | BHA2 | AR | Black | Yes | NND | - | 2 | 96 | - | - |
| BHA2_1A03 | **1081-01** | BHA2 | AR | Black | Yes | NDD | - | 7 | 91 | - | - |
| BHA2_1E02 | **1107-01** | BHA2 | AR | Brown | Yes | NDD | - | 11 | 87 | - | - |
| BHA2_1C05 | **1042-01** | BHA2 | AR | Straw | Yes | NND | - | 15 | 79 | 5 | 1 |
| BHA2_1E08 | **1202-02** | BHA2 | AR | Brown | Short | NND | 1 | 40 | 52 | 6 | 2 |
| BHA1_1B11 | StgB | BHA1 | AR | Black | Yes | NND | 6 | 70 | 21 | 3 | 1 |
| BHA1_1D07 | 1995-13 | BHA1 | LA | Black | Yes | NND | - | 83 | 13 | 2 | 1 |
| BHA1_1B02 | 10A | BHA1 | AR | Black | Yes | . | - | 84 | 14 | - | - |
| BHA1_1B08 | 1996-09 | BHA1 | MS | Black | Yes | NND | - | 84 | 15 | - | - |
| BHA1_1E09 | **2002-2-p1** | BHA1 | AR | Black | Yes | NND | - | 88 | 2 | 2 | 7 |
| BHA1_1D08 | 1995-14 | BHA1 | LA | Straw | Yes | DDN | - | 97 | 1 | 1 | - |
| BHA1_1B09 | LA3 | BHA1 | LA | Straw | Yes | DDD | - | 97 | 1 | 1 | - |
| BHA1_1B12 | StgS | BHA1 | AR | Straw | Yes | DDD | - | 97 | 1 | 1 | - |
| BHA1_1D05 | PrCoTall_3 | BHA1 | AR | Black | Yes | NND | - | 98 | - | - | - |
| BHA1_1E01 | **1166-02** | BHA1 | MS | Black | Yes | NND | - | 98 | - | - | - |
| BHA1_1A05 | **1096-01** | BHA1 | AR | Black | Yes | NND | - | 98 | - | - | - |
| BHA1_1B04 | 18A | BHA1 | AR | Black | Yes | NND | - | 98 | - | - | - |
| BHA1_1C04 | **1005-02** | BHA1 | AR | Black | Yes | NND | - | 98 | - | - | - |
| BHA1_1D02 | PrCoSrt_1 | BHA1 | AR | Black | Yes | NND | - | 98 | - | - | - |
| BHA1_1D03 | PrCoTall_1 | BHA1 | AR | Straw | Yes | NND | - | 98 | - | - | - |
| BHA1_1D04 | PrCoTall_2 | BHA1 | AR | Straw | Yes | NND | - | 98 | - | - | - |

^a^ Accessions in bold were selfed 4 generations at the USDA stock center

^b^ Population codes for weedy rice clusters identified by InStruct see text for details.

^c^ Three cytoplasmic markers combined into single cytotype and listed in the order ORF100, SSV500, SSV36; D - deletion; N - no deletion

^d^ Proportion of individual’s genome with ancestry in weedy population listed in column. - Membership proportion approximately zero.
