# Supplementary material for: Genomic patterns of nucleotide diversity in divergent populations of U.S. weedy rice
Source: BMC Evol Biol. 2010 Jun 15;10:180. doi: 10.1186/1471-2148-10-180 (PMC2898691; doi:10.1186/1471-2148-10-180)
Supplement: Additional file 4 — Supplementary text 1. MS command line and description of parameter values. [file 1471-2148-10-180-S4.DOC]

**IMA assumptions**

IMa assumes that the demographic history of a population pair can be modeled as two populations splitting from an ancestor, which then exchange migrants only with each other. IMa also assumes that inter-locus recombination occurs freely and that no intra-locus recombination has occurred since divergence. We considered this likely, based on the large distances separating STS on the same chromosome (Additional file 3), and the reduction of effective recombination rates within loci characteristic of highly selfing species (Nordburg and Donnelly, 1997) coupled with recent divergence. We assumed that our randomly sampled STS fragments were neutral; however, previous studies have reported that IMa is fairly robust to violations of no recombination and selective neutrality (Hey and Nielson, 2007; Strasburg and Rieseberg, 2008). For two of the three population pairs compared, a single STS fragment was excluded from the analysis due to occurrence of a triallelic SNP; we thus used the infinite sites mutation model for all simulations, as recommended in the IMa manual.

**References**

Hey J, Nielsen R: **Integration within the Felsenstein equation for improved Markov chain Monte Carlo methods in population genetics**. *Proceedings of the National Academy of Sciences* 2007, **104**(8):2785-2790.

Nordborg M, Donnelly P: **The coalescent process with selfing**. *Genetics* 1997, **146**(3):1185-1195.

Strasburg JL, Rieseberg LH: **Molecular demographic history of the annual sunflowers Helianthus annuus and H. petiolaris; large effective population sizes and rates of long-term gene flow**. *Evolution* 2008, **62**(8):1936-1950.

**MS Command line:**

./ms **INDS** **SIMS** **-t** θ_N_ **-r** θ_N_ 48 **-I** 2 22 24 **-n** 2 η_r_ **-en** τ_g_ 2 η_b_ **-en** τ_f_ 2 η_c_ -**ej** τ_s_ 2 1

**Explanation of code**

**INDS.**  number of copies of locus in sample

**SIMS.** number of simulations (independent genealogies)

**-I** 2 22 24 simulate 2 populations with 22 individuals in pop. 1 (η_c_ = *O. sativa indica* in this example) and 24 individuals in pop. 2 (η_r_ = SH weedy rice)

**-t** θ_N_ θ_N_ = (θ_wηc_ /(θ_w_ *_O.rufipogon_*) * 62.303. The 62.303 was calculated from the point estimate of *O.rufipogon* population size in Caicedo et al. 2007.

**-r** θ_N_ 48. estimate of recombination rate set equal to θ_ref_; We pooled all loci and therefore assumed 48 mostly independent loci of equal sized fragments

**-I** 2 22 24 simulated 2 population with 22 individuals in pop. 1 (η_c_ = *O. sativa indica* in this example) and 24 individuals in pop. 2 (η_r_ = SH weedy rice)

**-n** 2 η_r_ current size of weedy rice population

**-en** τ_g_ 2 η_b_ τ_g_ time of population expansion in US; 2 weedy population; η_b_ bottleneck population size

**-en** τ_f_ 2 1 τ_f_ time of founding in the US; 2 weedy population; η_c_ population returns to η_c_

-**ej** τ_s_ 2 1 τ_s_ time of split in the US; weedy population; population remains at η_c_

**Parameter Values**

for construction of grid

θ_wηc_ /θ_w_ *_O.rufipogon_* = 0.10, 0.15, 0.20, 0.25, 0.30, 0.35, 0.40, 0.45, 0.50, 0.55, 0.60, 0.65, 0.70

τ_g_ = 30,50,75,100,125,150

τ_f_ = 30,50,75,100,125,150,200,250,300,350,400

τ_s_ = 1000, 3000,7000,12000, 25000,50000

η_r_/η_c_  random uniform 0 - 1

η_b_/η_r_ uniform on 0 - η_r_ for a given simulation
