# Supplementary material for: Genomic patterns of nucleotide diversity in divergent populations of U.S. weedy rice
Source: BMC Evol Biol. 2010 Jun 15;10:180. doi: 10.1186/1471-2148-10-180 (PMC2898691; doi:10.1186/1471-2148-10-180)
Supplement: Additional file 5 — Supplementary Table 4. Mean DIC (5 simulations) for InStruct analysis of A) only U.S. weedy rice (n = 58), B) all Oryza accessions (n = 209). [file 1471-2148-10-180-S5.DOC]

Supplementary Table 4. Mean DIC (5 simulations) for InStruct analysis of A) only U.S. weedy rice (n = 58), B) all *Oryza* accessions (n = 209).

A. B.

| *K* | DIC^†^ | ΔDIC^*^ |
| --- | --- | --- |
| 4 | 3257.75 | 304.31 |
| 5 | 3205.05 | 251.62 |
| 6 | 3093.22 | 139.79 |
| 7 | 3053.58 | 100.15 |
| 8 | 3001.36 | 47.92 |
| **9** | **2953.44** | **0.00** |
| 10 | 2969.79 | 16.35 |
| 11 | 2986.24 | 32.81 |
| 12 | 2984.47 | 31.03 |
| 13 | 2989.57 | 36.13 |
| 14 | 3016.36 | 62.92 |
| 15 | 3029.17 | 75.73 |

| K | DIC^†^ | ΔDIC^*^ |
| --- | --- | --- |
| 2 | 1001.42 | 295.05 |
| 3 | 816.70 | 110.33 |
| 4 | 774.83 | 68.46 |
| 5 | 743.24 | 36.87 |
| **6** | **706.37** | **0.00** |
| 7 | 719.92 | 13.56 |
| 8 | 733.93 | 27.56 |
| 9 | 745.80 | 39.43 |
| 10 | 758.25 | 51.88 |

† Lowest DIC value indicates best fitting model *K* = 9 in bold.

* ΔDIC absolute value of difference between DIC for each *K* and the minimum DIC differences greater than 5-10 are considered substantial.
