# Supplementary material for: Genomic patterns of nucleotide diversity in divergent populations of U.S. weedy rice
Source: BMC Evol Biol. 2010 Jun 15;10:180. doi: 10.1186/1471-2148-10-180 (PMC2898691; doi:10.1186/1471-2148-10-180)
Supplement: Additional file 6 — Supplementary Table 5. Number of segregating sites, fixed and shared polymorphisms and private sites between various Oryza groups. [file 1471-2148-10-180-S6.DOC]

Supplementary Table 5. Number of segregating sites, fixed and shared polymorphisms and private sites between various *Oryza* groups.

| Population^a^ | P^b^ | S^c^ | Fixed | Shared | Private_1^d^ | Private_2 |
| --- | --- | --- | --- | --- | --- | --- |
| BHA1 - *aus* | 31 | 95 | 1 | 27 | 15 (34)^e^ | 14 (33) |
| BHA2 - *aus* | 29 | 91 | 1 | 13 | 9 (9) | 25 (30) |
| SH - *indica* | 35 | 146 | 0 | 37 | 1 (4) | 51(105) |
| *aus* - *indica* | 39 | 165 | 2 | 39 | 10(22) | 55 (102) |
| BHA1-BHA2 | 23 | 77 | 0 | 22 | 21 (25) | 2 (7) |
| BHA1 – *O. rufipogon* | 48 | 652 | 1 | 57 | 3 (3) | 591 (521) |
| SH – *O. rufipogon* | 48 | 651 | 0 | 38 | 2 (3) | 540 (610) |
| *aus* – *O. rufipogon* | 48 | 645 | 0 | 54 | 2 (7) | 516 (584) |
| *indica* – *O. rufipogon* | 48 | 661 | 0 | 130 | 7 (12) | 448 (518) |

^a^ Population numbering is in order listed in the first column

^b^ Number of polymorphic STS loci for population pair

^c^ Total number of segregating sites for population pair

^d^ Number of non-singleton private SNPs; total number of private SNPs, including singletons are shown in parenthesis

^e^14 singleton SNPs are contributed by a single individual (1E09) in a single STS (sts071)
