# Supplementary material for: Genomic patterns of nucleotide diversity in divergent populations of U.S. weedy rice
Source: BMC Evol Biol. 2010 Jun 15;10:180. doi: 10.1186/1471-2148-10-180 (PMC2898691; doi:10.1186/1471-2148-10-180)
Supplement: Additional file 7 — Supplementary Table 6. ML estimates and 90% posterior density intervals (HPD) of demographic parameters for three population pairs. [file 1471-2148-10-180-S7.DOC]

Supplementary Table 6. ML estimates and 90% posterior density intervals (HPD) of demographic parameters for three population pairs

| Population Pair | ***N_e1_ ^a^*** | ***N_e2_ ^a^*** | ***N_eA_^b^*** | ***t^c^*** |
| --- | --- | --- | --- | --- |
| BHA1 – *aus*^d^ | 0.05 | 0.11 | 1.56 | 0.05 |
|  | (0.03-0.09) | (0.05-0.26) | (0.87-3.37) | (0.02-0.14) |
| SH - *indica* ^d^ | 0.02 | 0.32 | 1.49 | 0.16 |
|  | (0.006-0.038) | (0.19-0.61) | (0.72-4.90^*^) | (0.05-0.41) |
| *aus - indica* ^d^ | 0.09 | 0.26 | 0.86 | 0.03 |
|  | (0.04-0.23) | (0.09-0.39) | (0.40-4.90^*^) | (0.003-1.2) |

^a^ Effective population size (N_e_) in order listed (i.e. BHA1 is N_e1_ and *aus* is N_e2_), numbers in parenthesis represent the 90% posterior density intervals.

^b^ Ancestral population effective population size; numbers in parenthesis represent the 90% posterior density intervals.

^c^ Population split time; numbers in parenthesis represent 90% posterior density intervals. Asterisks indicate posterior density intervals that exceed maximum priors for the parameter.

^d^ number of loci used in analysis: BHA1-*aus* 27; SH-*indica* 28; *aus*-*indica* 32
