# Supplementary material for: Genomic patterns of nucleotide diversity in divergent populations of U.S. weedy rice
Source: BMC Evol Biol. 2010 Jun 15;10:180. doi: 10.1186/1471-2148-10-180 (PMC2898691; doi:10.1186/1471-2148-10-180)
Supplement: Additional file 8 — Supplementary Figure 1. Marginal posterior probability curves for demographic parameters for each population pair comparison as estimated by IMa. All estimates have not been converted to individuals or years A) ancestral population size for aus - indica, B) time of divergence aus - indica, C) migration from aus to indica in red, migration from indica to aus in black, D) population size of aus in red and indica in black, E) ancestral population size for aus - BHA1, F) time of divergence aus - BHA1, G) migration from aus to BHA1 in red, migration from BHA1 to aus in black, H) population size of aus in red and BHA1 in black, I) ancestral population size for indica - SH, J) time of divergence indica - SH, K) migration from indica to SH in red, migration from SH to indica in black, L) population size of indica in red and SH in black. [file 1471-2148-10-180-S8.PDF]

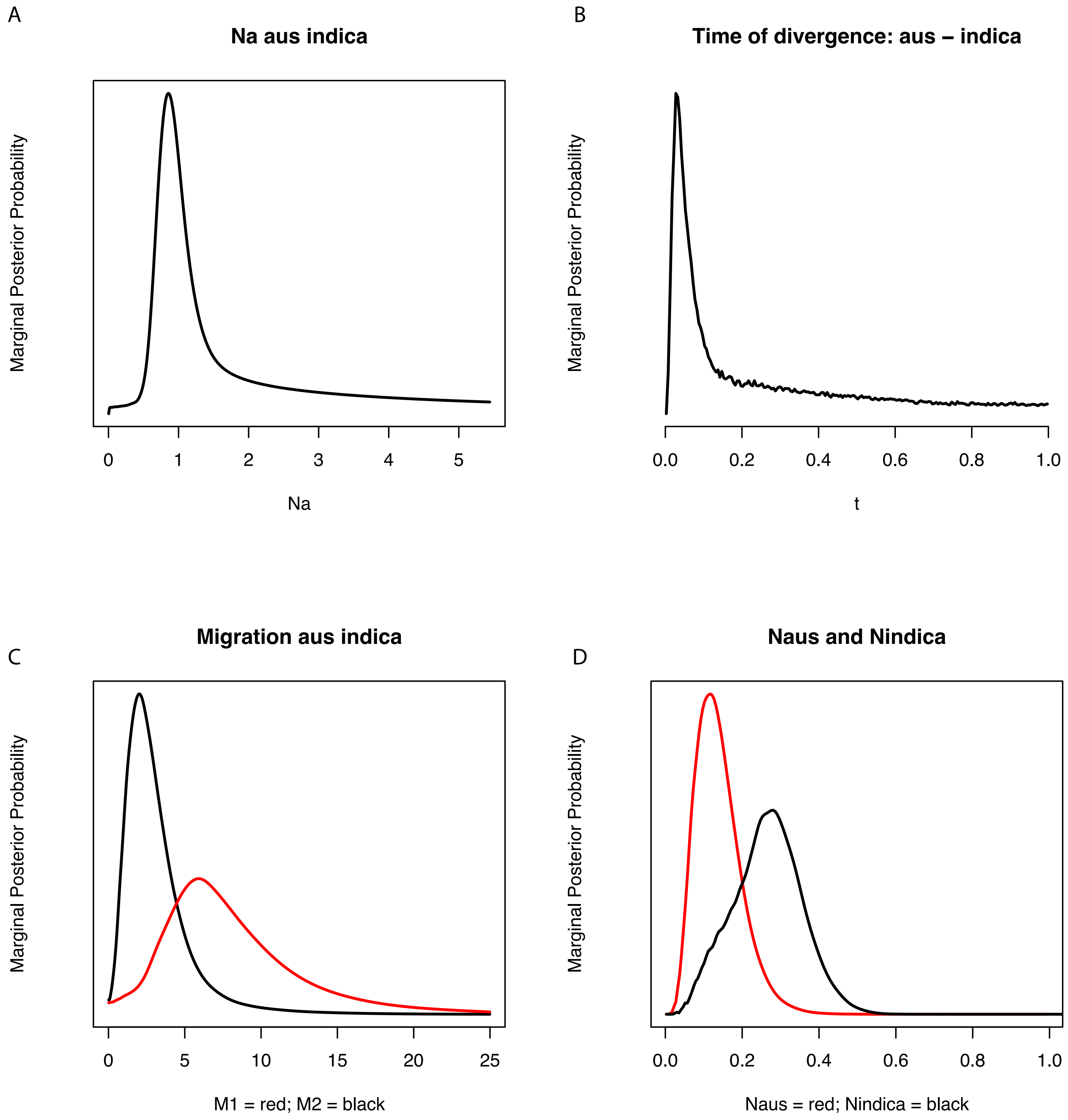
A
Na aus indica
Marginal Posterior Probability
0
1
2
3
4
5
Na
B
Time of divergence: aus – indica
Marginal Posterior Probability
0.0
0.2
0.4
0.6
0.8
1.0
t
C
Migration aus indica
Marginal Posterior Probability
0
5
10
15
20
25
M1 = red; M2 = black
D
Naus and Nindica
Marginal Posterior Probability
0.0
0.2
0.4
0.6
0.8
1.0
Naus = red; Nindica = black

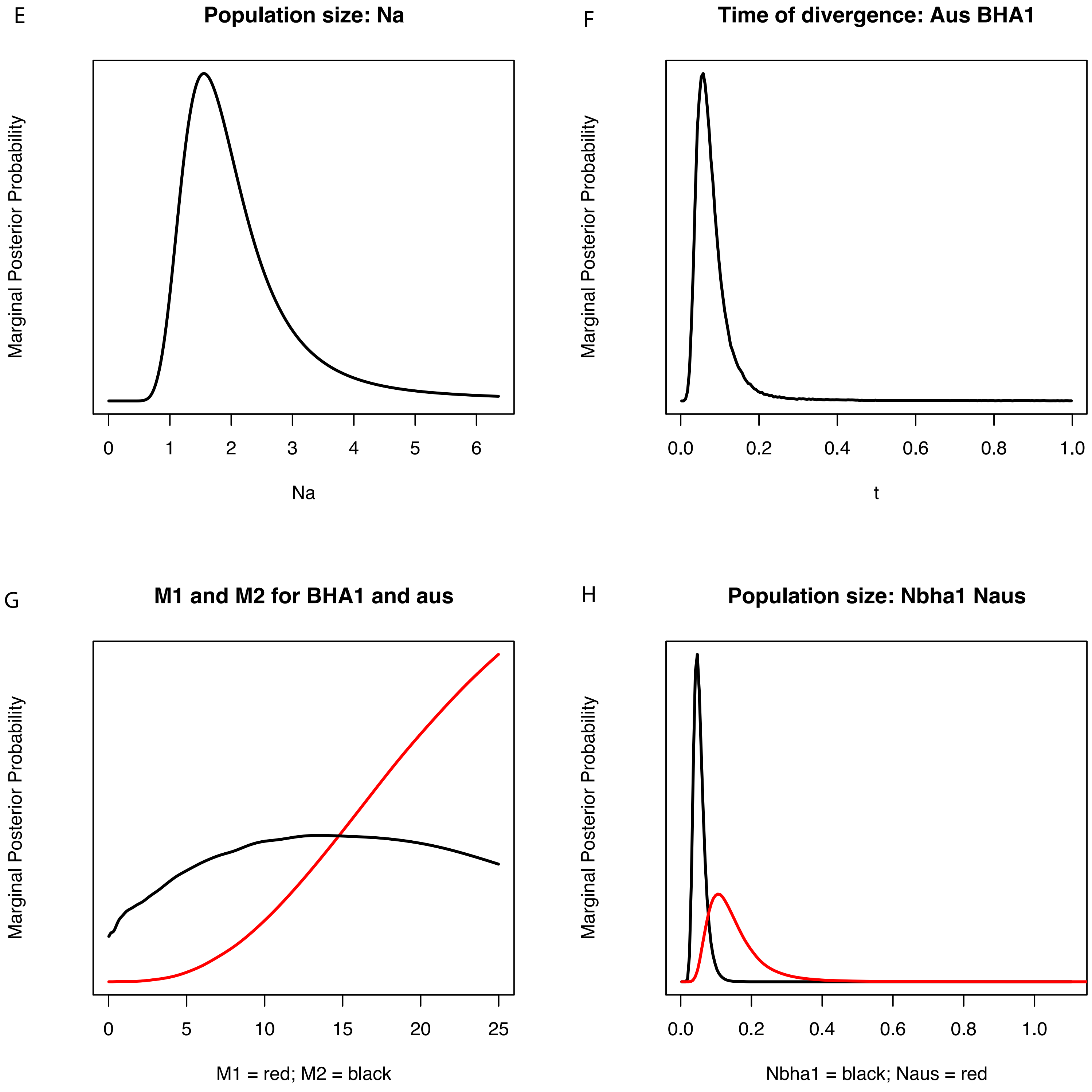
E
Population size: Na
Marginal Posterior Probability
0
1
2
3
4
5
6
Na
F
Time of divergence: Aus BHA1
Marginal Posterior Probability
0.0
0.2
0.4
0.6
0.8
1.0
t
G
M1 and M2 for BHA1 and aus
Marginal Posterior Probability
0
5
10
15
20
25
M1 = red; M2 = black
H
Population size: Nbha1 Naus
Marginal Posterior Probability
0.0
0.2
0.4
0.6
0.8
1.0
Nbha1 = black; Naus = red

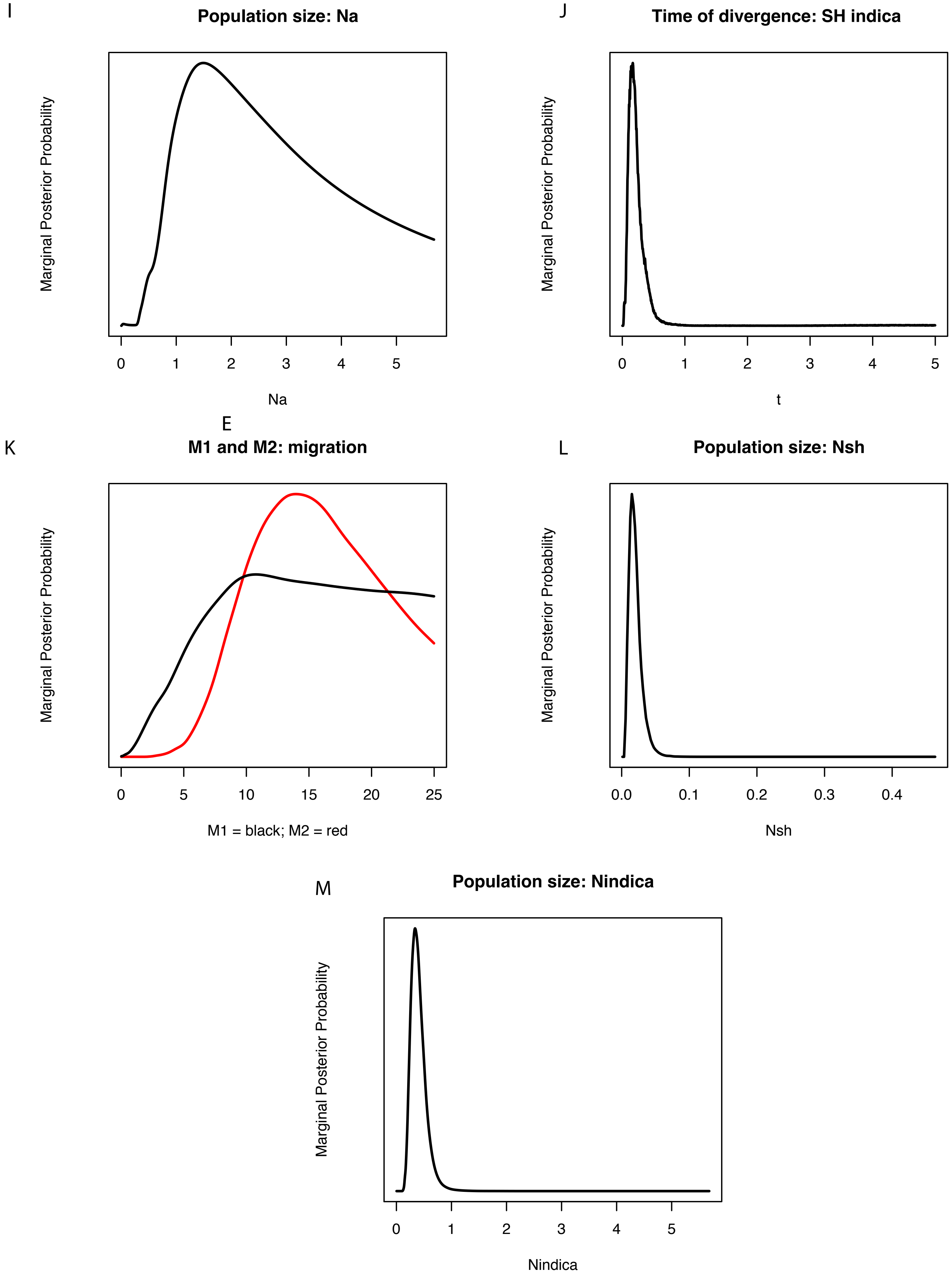
I
Population size: Na
Marginal Posterior Probability
0 1 2 3 4 5
Na
J
Time of divergence: SH indica
Marginal Posterior Probability
0 1 2 3 4 5
t
E
K
M1 and M2: migration
Marginal Posterior Probability
0 5 10 15 20 25
M1 = black; M2 = red
L
Population size: Nsh
Marginal Posterior Probability
0.0 0.1 0.2 0.3 0.4
Nsh
M
Population size: Nindica
Marginal Posterior Probability
0 1 2 3 4 5
Nindica
